# Real-World Treatment Patterns and Survival in Patients with Advanced Non-Small Cell Lung Cancer: An Italian Retrospective Cohort Study

**DOI:** 10.3390/cancers18030538

**Published:** 2026-02-06

**Authors:** Angelo Delmonte, Nicola Gentili, Andrea Roncadori, Roberta Maltoni, Valentina Danesi, Pooja Hindocha, Cátia Leal, Stavros Oikonomou, Marta Mella, Sarah Lay-Flurrie, Gabrielle Emanuel, Caroline Rault, Mrudula B. Glassberg, Adam Lee, Yong Yuan, Ilaria Massa

**Affiliations:** 1 Department of Medical Oncology, IRCCS Istituto Romagnolo per lo Studio dei Tumori (IRST) “Dino Amadori”, 47014 Meldola, Italy; 2 Outcome Research, Healthcare Administration, IRCCS Istituto Romagnolo per lo Studio dei Tumori (IRST) “Dino Amadori”, 47014 Meldola, Italy; nicola.gentili@irst.emr.it (N.G.); andrea.roncadori@irst.emr.it (A.R.); roberta.maltoni@irst.emr.it (R.M.); valentina.danesi@irst.emr.it (V.D.); 3 IQVIA Ltd., London W2 1AF, UK; pooja.hindocha@iqvia.com (P.H.); sarah.lay-flurrie@iqvia.com (S.L.-F.); 4 IQVIA Ltd., 2740-266 Porto Salvo, Portugal; catia.leal@iqvia.com; 5 IQVIA Ltd., 1303 Sofia, Bulgaria; stavros.oikonomou@iqvia.com; 6 IQVIA Ltd., 20124 Milan, Italy; marta.mella@iqvia.com; 7 Bristol Myers Squibb, Uxbridge UB8 1DH, UK; 8 Data Gnosis, 35000 Rennes, France; caroline.rault@bms.com; 9 Bristol Myers Squibb, Madison, NJ 07940, USA; mrudula.glassberg@bms.com; 10 Bristol Myers Squibb, Princeton, NJ 08540, USA

**Keywords:** Onco-Optimise, real-world evidence, non-small-cell lung cancer (NSCLC), locally advanced, metastatic, treatment patterns, overall survival, progression-free survival, Italy

## Abstract

**Background:** Treatment for advanced non-small-cell lung cancer (NSCLC) has evolved substantially over the past decade, necessitating evaluation of real-world treatment patterns and effectiveness before and after the introduction of newer therapies. **Methods:** This retrospective cohort study included adult patients initiating a non-curative first-line therapy for advanced NSCLC between 2014 and 2021 at the IRCCS Istituto Romagnolo per lo Studio dei Tumori (IRST), with follow-up through 2022. Overall survival (OS) and real-world progression-free survival from the start date of the first line of therapy for advanced NSCLC were estimated using Kaplan–Meier methods. **Results:** Overall, 910 patients were included; at diagnosis, 83% had a de novo diagnosis and 17% had recurrent disease. During the study, 41% of patients received platinum-based chemotherapy alone; 22% received non-platinum-based chemotherapy alone; 21% received anti-programmed death (PD)-(ligand [L])1 immune checkpoint inhibitors (ICIs), alone or with chemotherapy; and 16% received targeted therapy (single-agent tyrosine kinase inhibitors) as first-line therapy for advanced disease. From 2014 to 2021, the proportion of patients receiving first-line anti-PD-(L)1 ICIs increased from 0% to 58% and the proportion of those receiving first-line platinum-based chemotherapy decreased from 65% to 6%. Median (95% CI) OS was 8.2 (7.5–9.2) months; the minimum-to-maximum range of median OS was 6.0–7.4 months from 2014 to 2017 and 8.4–15.9 months from 2018 to 2021. Median OS was numerically longer in patients with recurrent disease versus a de novo diagnosis, first-line targeted versus other therapy, high versus low PD-L1 expression, and non-squamous/other versus squamous histology. **Conclusions:** This study provides real-world data further supporting (i) the benefits of precision targeted therapy for patients with advanced NSCLC and actionable genomic alterations and (ii) the positive impact of immunotherapy approvals on the treatment paradigm for advanced NSCLC.

## Simple Summary

Globally, lung cancer is the leading cause of cancer-related mortality, with most patients diagnosed with advanced (stage III or IV) non-small-cell lung cancer (NSCLC). Available treatments for advanced NSCLC have significantly expanded over the past decade, creating a need to evaluate real-world treatment patterns and effectiveness before and after the introduction of these newer therapies. This study describes patient characteristics, treatment patterns and survival among patients with advanced NSCLC (including locally advanced or metastatic disease) at the IRCCS Istituto Romagnolo per lo Studio dei Tumori (IRST) in Italy between 2014 and 2022. In alignment with other real-world studies, results from this study suggest that, despite a generally poor prognosis for patients with advanced NSCLC, those with actionable genomic alterations benefit from treatment with precision targeted therapies, and the approval of immunotherapies has further evolved the treatment paradigm for advanced NSCLC.

## 1. Introduction

Lung cancer is the leading cause of cancer-related death worldwide, accounting for 19% of all cancer-related mortality [1]. Based on the latest GLOBOCAN data, there were approximately 44,000 new cases of lung cancer in Italy in 2022, representing 10% of all newly diagnosed cancers in the country. In alignment with global data, approximately 18% of all cancer-related deaths in Italy in 2022 were attributed to lung cancer [2]. Approximately 90% of lung cancer cases are non-small-cell lung cancer (NSCLC) [3]. Globally, NSCLC is typically diagnosed at an advanced stage, with approximately 30% of patients diagnosed with locally advanced disease (stage III) and around 50% with metastatic disease (stage IV) [4]. In a recent Italian real-world study of patients with NSCLC, 21% had stage III disease and 51% had stage IV disease at diagnosis [5]. Historically, prognosis for patients with NSCLC has been poor, particularly in the advanced stages; based on data from analyses conducted in 2016 as part of the International Association for the Study of Lung Cancer (IASLC) International Staging Project, global 5-year overall survival (OS) rates were between 13% and 36% for patients with stage III disease, decreasing to <10% for those with stage IV disease [6].

Before the approval of first-line immunotherapy-based regimens, the European Society for Medical Oncology (ESMO) recommended platinum-based chemotherapy as initial treatment for fit patients (i.e., without major comorbidities and with a performance status [PS] of 0–2) with advanced NSCLC without actionable genomic alterations [7]. The identification of actionable genomic alterations within lung cancer cells, such as those in the epidermal growth factor receptor (*EGFR*), anaplastic lymphoma kinase (*ALK*), B-rapidly accelerated fibrosarcoma (*BRAF*) and c-ros oncogene 1 receptor tyrosine kinase (*ROS1)* genes, has led to the development of numerous novel targeted therapies for patients with NSCLC, highlighting the importance of molecular profiling as part of the overall NSCLC diagnostic profile [8]. Moreover, the treatment landscape for patients with advanced NSCLC without actionable genomic alterations has also evolved with the approval of immunotherapies, in particular, immune checkpoint inhibitors (ICIs) targeting the programmed death-1/programmed death ligand 1 (PD-[L]1) pathway (e.g., pembrolizumab, atezolizumab, nivolumab, durvalumab) [9,10,11,12,13]. Due to these therapeutic advances, ESMO guidelines now recommend a plethora of systemic anti-cancer therapy (SACT) regimens for patients with advanced NSCLC, including chemotherapy, targeted therapies and immunotherapy, either alone or in a variety of combinations [14,15]. For most patients with advanced NSCLC without actionable genomic alterations, ICIs are now considered to be the first-line standard of care [14,16]. In Italy, nivolumab was the first ICI to be reimbursed for patients with NSCLC, receiving approval from the Italian Medicines Agency (AIFA) for the second-line treatment of patients with locally advanced or metastatic squamous cell NSCLC in 2016 and then in 2017 for locally advanced or metastatic non-squamous cell NSCLC [17,18]. Pembrolizumab was subsequently approved by AIFA in 2017 for both first- and second-line treatment of patients with advanced NSCLC [19], atezolizumab was approved in 2018 for second-line treatment of patients with locally advanced or metastatic NSCLC [20], and nivolumab in combination with ipilimumab and two cycles of platinum chemotherapy was approved in December 2021 for the first-line treatment of patients with metastatic NSCLC without *EGFR* or *ALK* mutations [21].

Given the rapidly evolving treatment landscape for patients with advanced NSCLC, there is a need to evaluate the effectiveness of newer treatments outside of clinical trial settings and to understand treatment patterns and outcomes before and after the introduction of these newer therapies. Onco-Optimise (formerly known as I-O Optimise) is an ongoing, international collaborative research initiative leveraging existing real-world cancer data sources to provide insights into the management of thoracic malignancies in clinical practice [22,23]. As part of Onco-Optimise, the current study was designed to retrospectively describe patient characteristics, treatment patterns, and survival outcomes among patients diagnosed with advanced NSCLC at the IRCCS Istituto Romagnolo per lo Studio dei Tumori (IRST) “Dino Amadori” of Meldola (Forlì-Cesena) in Italy, before and after the availability of newer treatment options for advanced NSCLC.

## 2. Materials and Methods

### 2.1. Eligibility Criteria and Study Design

This was a single-centre, retrospective cohort study using data from electronic health records (EHR; CCE Log80 2.6 of Log80 S.r.l) collected at the IRST for patients with advanced NSCLC. The IRST is a centre of excellence entirely dedicated to treatment, research, and training in the oncology field. Established in 2007 and situated in Meldola, a small town in Emilia-Romagna in the Forlì-Cesena province of Italy, the IRST is fully integrated within the Italian National Health Service (Servizio Sanitario Nazionale). For the current study, patients were included if they (i) had initiated a non-curative first line of therapy for advanced NSCLC (including for locally advanced [stage III] or metastatic [stage IV] disease) between 1 January 2014 and 31 December 2021 as recorded in the IRST database, (ii) were residents in the Emilia-Romagna region and (iii) were ≥18 years at the date of advanced NSCLC diagnosis. The study period was chosen to capture both the pre-immunotherapy era and the subsequent period after approvals of first- and second-line immunotherapy for advanced NSCLC in Italy (e.g., nivolumab in 2016/2017 [17,18], pembrolizumab in 2017 [19], and atezolizumab in 2018 [20]). Patients who had missing data when determining eligibility, had no confirmed NSCLC diagnosis, received only curative therapy, never started SACT, received first-line therapy at another institution, or enrolled in clinical trials at the IRST at any time after their NSCLC diagnosis were excluded. Patients with any previous primary malignancy (excluding non-melanoma skin cancer; International Statistical Classification of Diseases and Related Health Problems 10th Revision [ICD-10] code C44) in the 5 years before initial NSCLC diagnosis were also excluded. Eligibility criteria were verified via manual review of patient information by an oncologist at the IRST. Patients were followed from the date they started their first non-curative line of therapy (i.e., their inclusion date) until the earliest of death, exit from the data source or the end of the study period (31 December 2022). Analyses were stratified according to diagnosis type at the inclusion date (de novo diagnosis or recurrent disease). A de novo diagnosis refers to patients starting a first line of therapy after an incident diagnosis of advanced NSCLC; recurrent disease refers to patients receiving either a previous NSCLC diagnosis at an earlier disease stage or prior curative treatment before starting a first line of therapy.

### 2.2. Variables

Demographic and clinical characteristics were collected for all patients at the time of diagnosis of advanced NSCLC and/or their inclusion date. Data collection was followed by data cleaning and supplemented by a manual review of unstructured data (i.e., clinical notes, radiology and pathological reports) to achieve a high completion rate for this analysis. Histology at diagnosis (defined according to the International Classification of Diseases for Oncology—2nd edition [ICD-O-2] morphology codes) was categorised as either “non-squamous NSCLC and other” (including adenocarcinoma, large cell carcinoma, carcinoma not otherwise specified and others) or “squamous NSCLC”. Tumour–node–metastasis (TNM) staging followed the American Joint Committee on Cancer (AJCC)/Union for International Cancer Control (UICC) Staging Manual (7th or 8th edition) and was categorised according to the recommended edition at the time of the patient’s inclusion date. In relation to testing for tumour PD-L1 expression and key actionable genomic alterations, the designated categories were as follows: “conclusive test result” (i.e., patients with quantifiable PD-L1 expression data or a confirmed positive or negative test result for *EGFR*, *ALK* or *ROS1* mutations) or “inconclusive test result/not tested” (i.e., patients who were tested but had inconclusive test results, or who were not tested for the respective parameter). Tumour PD-L1 expression was classified as low (<1% or negative), medium (1–49%) or high (≥50%) in patients with a conclusive test result.

Treatment sequencing was defined by the SACT regimens received at each line of therapy and the sequence of treatment across the different lines of therapy. Line of therapy was defined algorithmically as one or more SACT regimens (≥1 cycle of SACT administered). As line of therapy was not directly recorded at the IRST, an algorithm was used to retrieve line of therapy information from the EHR including the numbering of the lines of therapy and their start and end dates, as described previously [24]. Advancement of line of therapy was not influenced by maintenance therapy, interruptions or the replacement of any drug in the combination regimen with another due to toxicity. Discontinuation of the line of therapy was defined as the recorded date of treatment discontinuation or, if not recorded, the date of treatment at last administration if the gap between the last administration date and the next treatment administration was two times the treatment duration.

Additionally, for patients with stage IIIA/IIIB disease, a related treatment intent algorithm was also used (Table S1). If the first treatment recorded had curative intent, then the first SACT after the curative intent treatment was defined as the first line of therapy for advanced NSCLC, and if the first treatment recorded had advanced intent, then this treatment was considered the first line of therapy.

The type of therapy received was categorised as follows: “platinum-based chemotherapy” (included singlet, doublet, etc., platinum-based chemotherapy regimens, but not combinations of platinum-based chemotherapy with other classes of treatment), “non-platinum-based chemotherapy” (included singlet or doublet non-platinum-based chemotherapy regimens, but not combinations of non-platinum-based chemotherapy with other classes of treatment), “anti–PD-(L)1 ICI monotherapy” (included single-agent ICIs), “anti-PD-(L)1 ICIs + chemotherapy” (included combinations of ICIs with chemotherapy, but not combinations of ICIs), “targeted therapy” (included single-agent tyrosine kinase inhibitors [TKIs]) or “other treatments” (included combinations of TKIs or other monoclonal antibodies with other treatments [e.g., chemotherapy]). Surgery and/or radiotherapy were not captured in the treatment patterns analysis.

Using the line of therapy algorithms, treatment sequences (first through fourth line) were derived from the start date of first treatment until the start date of a new agent not included in the previous therapy regimen or death, whichever occurred first. Treatment duration in each line of therapy was calculated from the start date of first treatment administration until the end date of last administration. Discontinuation (i.e., end of treatment duration) was considered if there was a recorded date of treatment discontinuation, usually associated with a record of the reason for discontinuation, e.g., toxicity, progression or death. Data for patients who were alive but who did not start a new agent were manually checked to verify the correct discontinuation date.

Analysed survival outcomes were OS and real-world progression-free survival (rwPFS); rwPFS was defined as the time from inclusion date to the earliest of (i) the date of a record of progressive disease (registered by the physician during disease assessment visits), (ii) the start date of a new agent that was not included in the previous therapy regimen, or (iii) the date of death from any cause. For both OS and rwPFS, patients without an event were censored at the earliest of the end of the study period or the date of loss to follow-up.

### 2.3. Statistical Analysis

Patient demographic and clinical characteristics (age, sex, tumour stage, year of first-line therapy initiation, Eastern Cooperative Oncology Group (ECOG) PS, histology, recorded metastases, tumour PD-L1 expression and *EGFR*, *ALK* and *ROS1* mutational status) were described using summary statistics. Survival outcomes (i.e., OS and rwPFS) were estimated from the inclusion date using the Kaplan–Meier method, with the median value and corresponding 95% confidence intervals (CIs) presented. Treatment sequences are displayed using Sankey diagrams. Primary data masking was performed if patient counts for individual categories were between 1 and 4. Secondary data masking was performed, when necessary, to prevent calculation of primary masked data. To further prevent calculation of masked data, all percentages were rounded to the nearest whole number. No imputation methods were used to manage missing data except for date variables, which had missing dates imputed using the middle date of the month (15th).

### 2.4. Ethics

This study was conducted in accordance with the International Society for Pharmacoepidemiology (ISPE) Guidelines for Good Pharmacoepidemiology Practices (GPP) and applicable regulatory requirements. The study was approved by the Scientific and Medical Committee and the Ethic Committee of the IRST IRCCS Area Vasta Romagna (approval number: 3366). Informed patient consent was not required due to the retrospective nature of the study. All data are presented in aggregate form only.

## 3. Results

### 3.1. Patient Population

**Figure 1:**
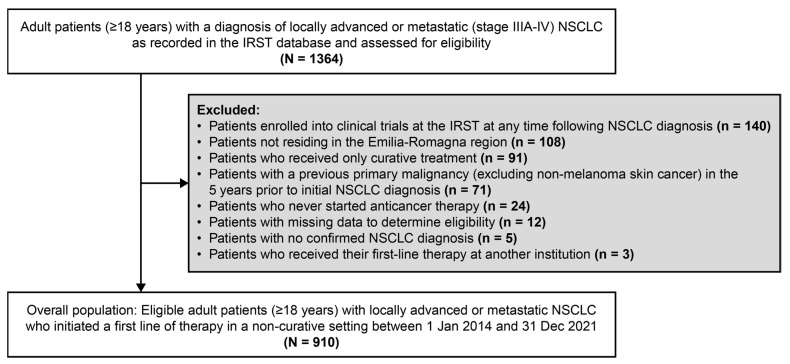
Patient flow chart. IRST: IRCCS Istituto Romagnolo per lo Studio dei Tumori; NSCLC: non-small-cell lung cancer.

Overall, 910 patients were included (Figure 1). Among them, 758 patients (83%) had a de novo diagnosis and 152 (17%) had recurrent disease. The median duration of follow-up from the inclusion date (interquartile range [IQR]) for the overall population was 10.6 (4.8–24.1) months and was longer among patients with recurrent disease versus a de novo diagnosis (21.1 [9.6–41.5] months versus 9.2 [4.3–21.9] months). Median age (IQR) was 69.9 (63.3–76.0) years, and almost all patients had stage IV disease (98%) (Table 1). Most patients had an ECOG PS of 0 or 1 (79%), with 16% having an ECOG PS of 2 and 2% an ECOG PS of 3. The most common metastatic sites were bone (26%) and contralateral lung (26%) (Table 1).

Characteristics were generally similar among patients with a de novo diagnosis and those with recurrent disease; the main differences were observed for ECOG PS (a higher proportion of patients with a de novo diagnosis than with recurrent disease had a EGOG PS of ≥2 [21% versus 6%]) and recorded metastatic sites (a higher proportion of patients with a de novo diagnosis than with recurrent disease had metastases in the bone [28% versus 16%], lymph nodes [18% versus 11%] and other sites [18% versus 7%]) (Table 1).

### 3.2. Testing Patterns

During the study, more than half of the patients (56%) had a conclusive tumour PD-L1 test result; 40%, 35% and 25% of these patients had low, medium and high PD-L1 expression, respectively (Table 1). The proportion of patients with a conclusive PD-L1 test result increased noticeably between 2016 and 2018. Of patients initiating a first line of therapy for advanced NSCLC in 2016, 9% had conclusive PD-L1 test results; by 2018, 89% of patients initiating a first line of therapy had a conclusive PD-L1 test result (Table S1). More than half of the patients also had a conclusive test result for the presence of actionable genomic alterations in the *EGFR* (77%), *ALK* (71%) or *ROS1* (52%) genes. Among patients with a conclusive test result, positive results were reported for 17%, 6% and 1% of patients, respectively (Table 1). The proportions of patients with a conclusive test result for the presence of *ALK* or *ROS1* mutations increased over the study period. Of the patients initiating a first line of therapy for advanced NSCLC in 2014, 43% had conclusive test results for *ALK* mutations and 10% had conclusive test results for *ROS1* mutations; by 2021, the proportions with conclusive test results were 81% and 80%, respectively (Table S2).

### 3.3. Treatment Patterns

Overall, platinum-based chemotherapy was the most frequently received first-line therapy for advanced NSCLC (41%), followed by non-platinum-based chemotherapy (22%), an anti-PD-(L)1 ICI (either as monotherapy or with chemotherapy; 21%) and targeted therapy (16%) (Table 2). Patients with a de novo diagnosis were more frequently treated with first-line platinum-based chemotherapy than those with recurrent disease (44% versus 22%). Conversely, patients with recurrent disease were more frequently treated with first-line anti-PD-(L)1 ICI monotherapy (27% versus 11%) or targeted therapy (20% versus 16%) than those with a de novo diagnosis (Table 2). Median treatment duration (IQR) for first-line therapy in the overall population was 13.4 (7.2–30.4) months; for patients with a de novo diagnosis, it was 13.2 (6.7–30.1) months, and for patients with recurrent disease, it was 15.0 (8.4–38.2) months (Table 2).

Among patients receiving first-line anti-PD-(L)1 ICIs for advanced NSCLC, 64% received ICI monotherapy and 36% received an ICI in combination with chemotherapy (mostly pembrolizumab with pemetrexed and carboplatin). In total, 38% of patients received an anti-PD-(L)1 ICI (either as monotherapy or with chemotherapy) at any point during the study period (Table 2). Most of these patients (56%) received the anti-PD-(L)1 ICI at the first line of therapy, with 38% receiving the anti-PD-(L)1 ICI at the second line of therapy and 6% at the third or a later line of therapy. No patients were retreated with an anti-PD-(L)1 ICI. Notably, of the patients with high tumour PD-L1 expression (i.e., PD-L1 ≥50%), 76% received first-line anti-PD-(L)1 ICI monotherapy. Of the patients receiving first-line targeted therapy, 72% were positive for *EGFR* mutations and 15% were positive for *ALK* mutations. Around 2–4% were positive for *ROS1* mutations, but the exact proportion could not be calculated due to masked data.

**Figure 2:**
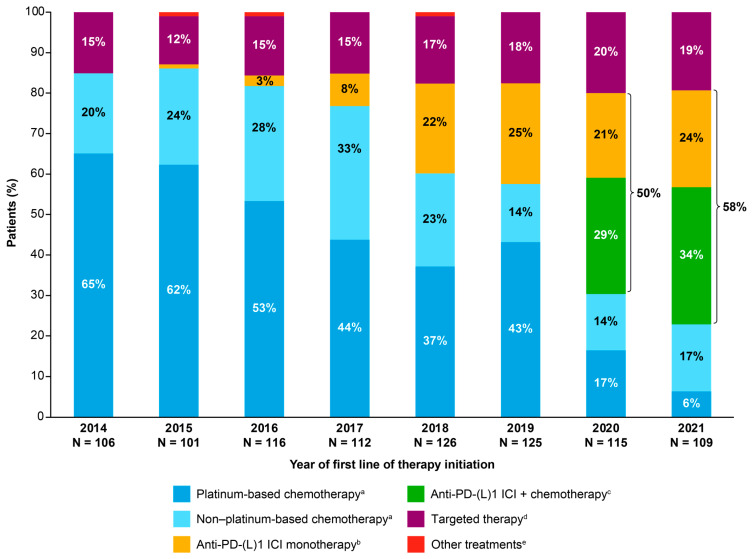
First-line treatment regimens in the overall population stratified by start year of first line of therapy for advanced NSCLC. Only proportions > 5% are noted in the chart. ^a^ Includes singlet, doublet, etc., chemotherapy regimens; does not include combinations of chemotherapy with other classes of treatment. ^b^ Includes single-agent anti-PD-(L)1 ICIs. ^c^ Includes combinations of anti-PD-(L)1 ICIs with chemotherapy; does not include combinations of anti-PD-(L)1 ICIs. ^d^ Includes single-agent TKIs. ^e^ Includes combinations of TKIs or other monoclonal antibodies with other classes of treatment (e.g., chemotherapy). ICI: immune checkpoint inhibitor; PD-(L)1: programmed death (ligand) 1; TKI: tyrosine kinase inhibitor.

There was a trend towards increased proportions of patients receiving first-line anti-PD-(L)1 ICI treatment (either as monotherapy or with chemotherapy) over the study period. In 2014, none of the patients initiating a first line of therapy for advanced NSCLC received an anti-PD-(L)1 ICI; by 2021, 58% of patients initiating a first line of therapy received an anti-PD-(L)1 ICI (Figure 2). In parallel, the proportion of patients receiving first-line platinum-based chemotherapy decreased from 65% in 2014 to 6% in 2021, and the proportion receiving first-line non-platinum-based chemotherapy decreased from 20% in 2014 to 17% in 2021. The proportion receiving targeted therapy increased marginally, from 15% in 2014 to 19% in 2021 (Figure 2).

**Figure 3:**
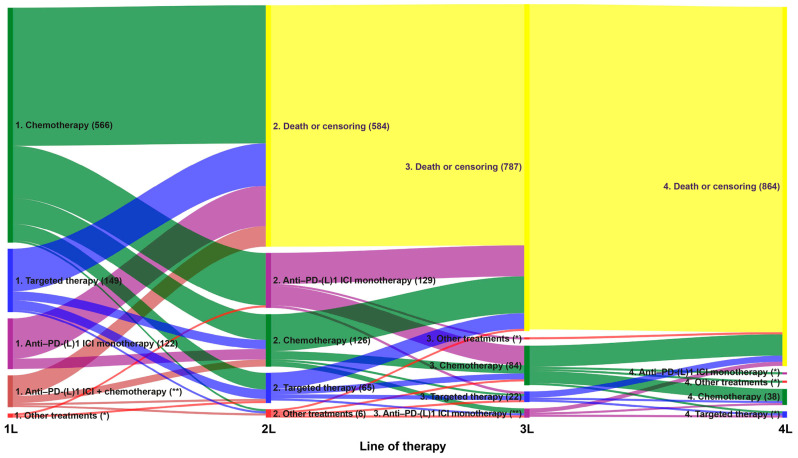
Treatment sequencing for the overall population. ICI: immune checkpoint inhibitor; PD-(L)1: programmed death (ligand) 1. * Indicates primary data masking of patient counts between 1 and 4; ** indicates secondary data masking to prevent calculation of primary masked data.

During the study, around two-thirds of patients (64%) received a single line of therapy for advanced NSCLC, 36% received at least two lines of therapy, 14% received at least three lines of therapy and only 5% received four or more lines of therapy (Table 2 and Figure 3). Treatment sequences from the first through fourth line of therapy are shown for the overall population in Figure 3. Of the patients who went on to receive a second line of therapy, 40% received anti-PD-(L)1 ICI monotherapy, 39% received chemotherapy (either platinum-based or non-platinum-based) and 20% received targeted therapy as their second line of therapy (Figure 3). Of the patients who went on to receive a third line of therapy, 68% received chemotherapy, 18% received targeted therapy and 13% received anti-PD-(L)1 ICI monotherapy as their third line of therapy (Figure 3). Among the small number of patients who went on to receive a fourth line of therapy, most (83%) received fourth-line chemotherapy (Figure 3). Treatment sequences for patients with a de novo diagnosis or recurrent disease are shown in Figures S1 and S2, respectively. Among patients who went on to receive a second line of therapy, a lower proportion of patients with a de novo diagnosis (38%) than with recurrent disease (46%) received anti-PD-(L)1 ICI monotherapy, and a higher proportion of patients with a de novo diagnosis (40%) versus recurrent disease (34%) received chemotherapy (either platinum-based or non-platinum-based) as their second line of therapy. Of the patients who went on to receive a third line of therapy, a lower proportion of patients with a de novo diagnosis (66%) than those with recurrent disease (77%) received chemotherapy (either platinum-based or non-platinum-based) as their third line of therapy.

### 3.4. Overall Survival

**Figure 4:**
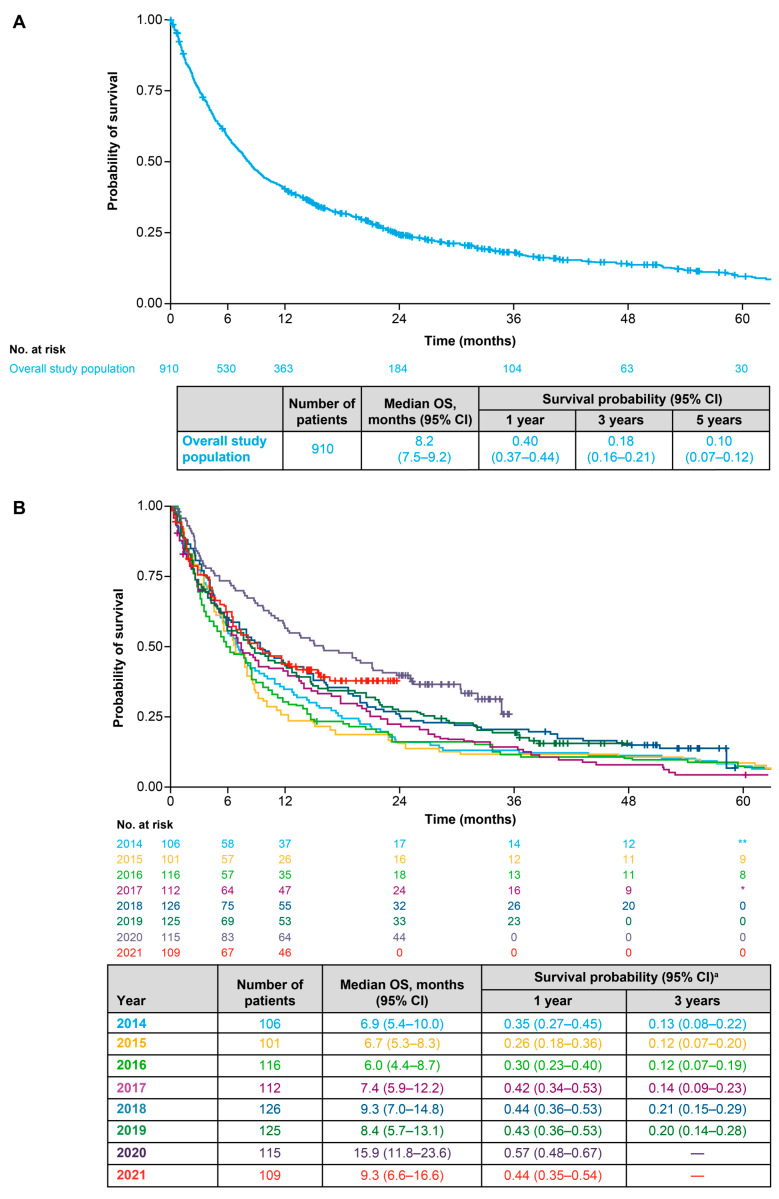
(**A**) Overall survival for the overall population. (**B**) Overall survival for overall population stratified by start year of first line of therapy for advanced NSCLC. ^a^ Survival probabilities are suppressed when the number of patients at risk is <10. CI: confidence interval; LoT: line of therapy; OS: overall survival. * Indicates primary data masking of patient counts between 1 and 4; ** indicates secondary data masking to prevent calculation of primary masked data.

**Figure 5:**
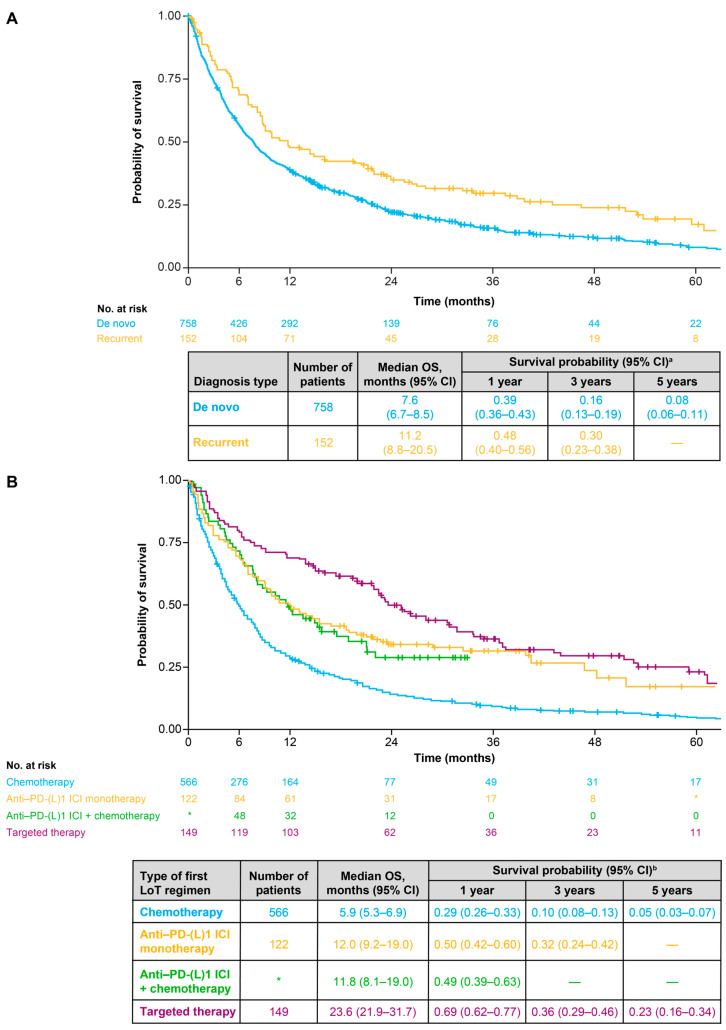
(**A**) Overall survival for the overall population stratified by diagnosis type (de novo diagnosis or recurrent disease). (**B**) Overall survival for the overall population stratified by type of first-line treatment for advanced NSCLC ^a^. * Indicates secondary data masking to prevent calculation of the number of patients in the “Other treatment” category. ^a^ “Other treatment” category could not be included due to small sample size (primary masked data). ^b^ Survival probabilities are suppressed when the number of patients at risk is <10. 1 L: first line; CI: confidence interval; ICI: immune checkpoint inhibitor; LoT: line of therapy; OS: overall survival; PD-(L)1: programmed death (ligand) 1.

Median OS (95% CI) in the overall population was 8.2 (7.5–9.2) months (Figure 4A). Between 2014 and 2017, the minimum-to-maximum (min–max) range of median OS was 6.0–7.4 months; between 2018 and 2021, the min–max range of median OS was 8.4–15.9 months (Figure 4B). Median OS (95% CI) was longer for patients with recurrent disease (11.2 [8.8–20.5] months) than for patients with a de novo diagnosis (7.6 [6.7–8.5] months) (Figure 5A). In terms of first-line treatments, median OS (95% CI) was 23.6 (21.9–31.7) months for patients receiving targeted therapy, 12.0 (9.2–19.0) months for those receiving anti-PD-(L)1 ICI monotherapy, 11.8 (8.1–19.0) months for those receiving an anti-PD-(L)1 ICI with chemotherapy and 5.9 (5.3–6.9) months for patients receiving any chemotherapy alone (either platinum-based or non-platinum-based) (Figure 5B). Median OS (95% CI) was 9.4 (7.0–14.7) months for patients with low PD-L1 expression, 10.6 (8.2–14.5) months for patients with medium PD-L1 expression and 15.5 (11.9–31.7) months for patients with high PD-L1 expression (Figure S3). Median OS (95% CI) was 8.5 (7.7–9.7) months for patients with non-squamous cell or other histology and 5.8 (5.2–8.8) months for patients with squamous cell histology (Figure S4).

### 3.5. Real-World Progression-Free Survival

**Figure 6:**
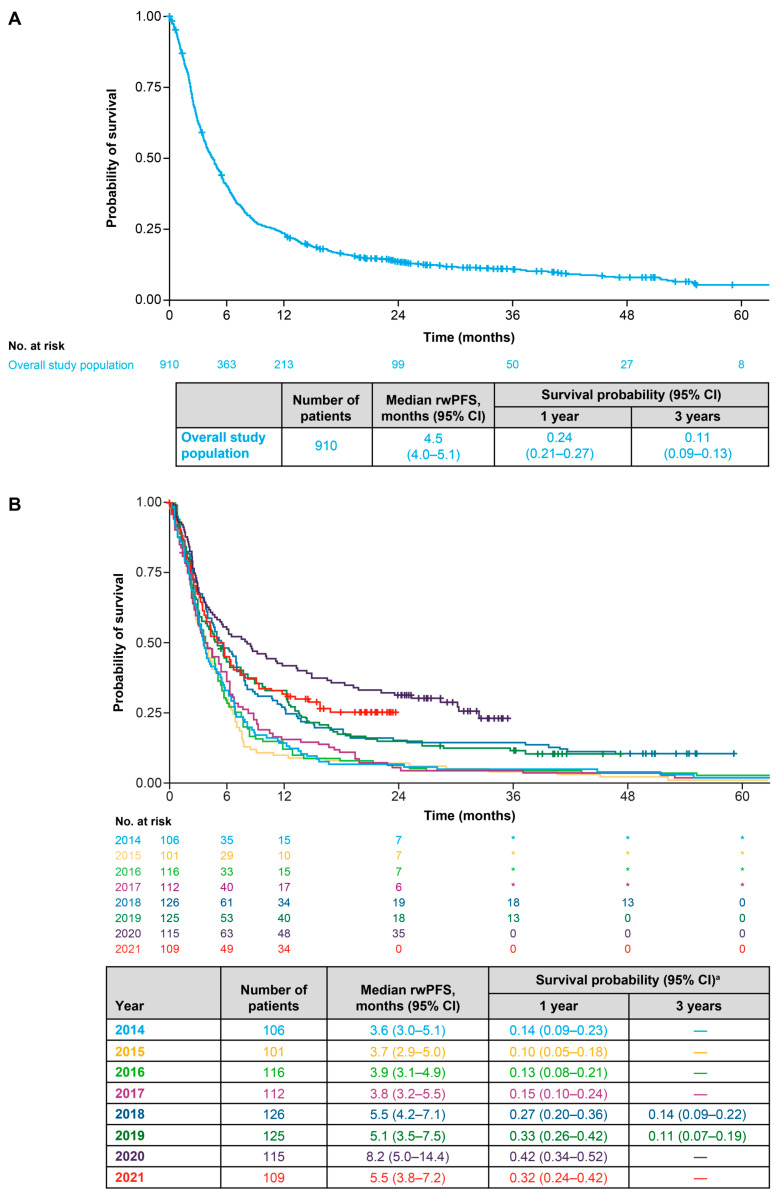
(**A**) Real-world progression-free survival for the overall population. (**B**) Real-world progression-free survival for the overall population stratified by start year of first line of therapy for advanced NSCLC. ^a^ Survival probabilities are suppressed when the number of patients at risk is <10. CI: confidence interval; rwPFS: real-world progression-free survival; * Indicates primary data masking of patient counts between 1 and 4.

**Figure 7:**
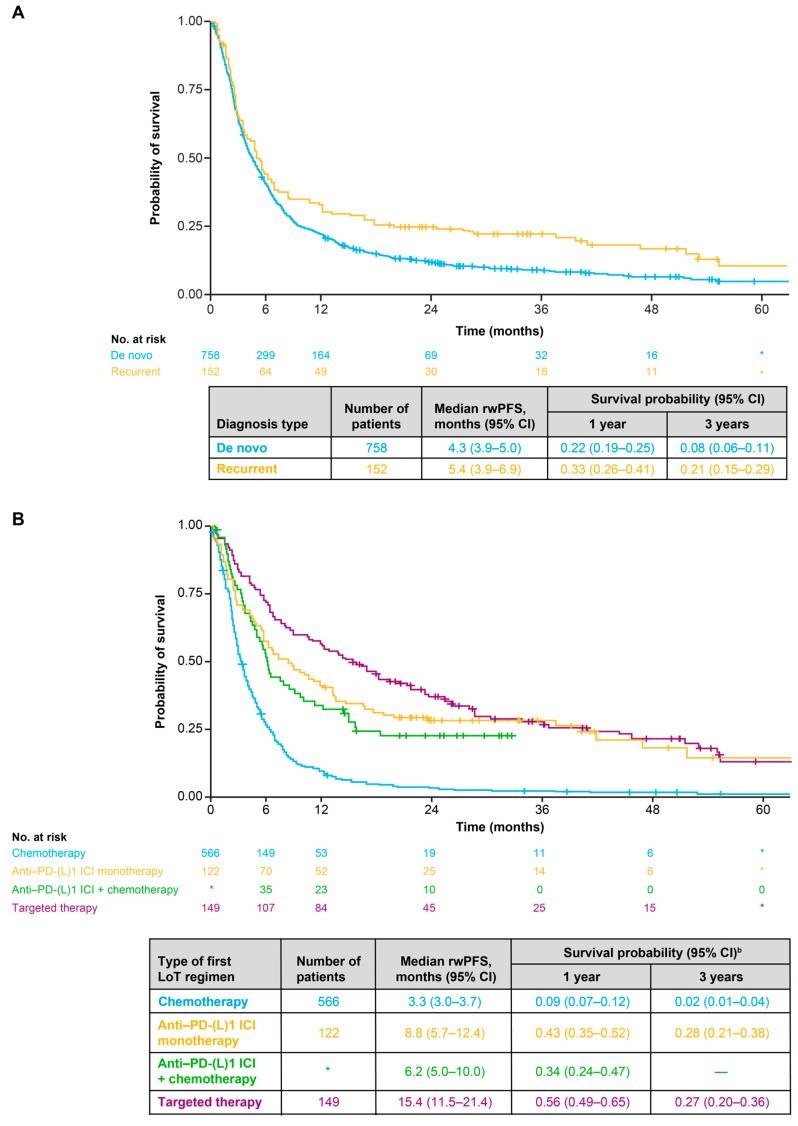
(**A**) Real-world progression-free survival for the overall population stratified by diagnosis type (de novo diagnosis or recurrent disease). (**B**) Real-world progression free survival for the overall population stratified by type of first-line treatment for advanced NSCLC ^a^. * Indicates secondary data masking to prevent calculation of the number of patients in the “Other treatment” category. ^a^ “Other treatment” category could not be included due to small sample size (primary masked data). ^b^ Survival probabilities are suppressed when the number of patients at risk is <10. 1 L: first line; CI: confidence interval; ICI: immune checkpoint inhibitor; LoT: line of therapy; PD-(L)1: programmed death (ligand) 1; rwPFS: real-world progression-free survival.

Median rwPFS (95% CI) in the overall population was 4.5 (4.0–5.1) months (Figure 6A). Between 2014 and 2017, the min–max range of median rwPFS was 3.6–3.9 months, and between 2018 and 2021, it was 5.1–8.2 months (Figure 6B). Median rwPFS (95% CI) was longer for patients with recurrent disease (5.4 [3.9–6.9] months) than for patients with a de novo diagnosis (4.3 [3.9–5.0] months) (Figure 7A). In terms of first-line treatments, median rwPFS (95% CI) was 15.4 (11.5–21.4) months for patients receiving targeted therapy, 8.8 (5.7–12.4) months for those receiving anti-PD-(L)1 ICI monotherapy, 6.2 (5.0–10.0) for those receiving an anti-PD-(L)1 ICI with chemotherapy and 3.3 (3.0–3.7) months for patients receiving any chemotherapy (Figure 7B). Median rwPFS (95% CI) was 4.3 (3.5–5.6) months for patients with low PD-L1 expression, 5.5 (4.7–6.6) months for patients with medium PD-L1 expression and 9.8 (6.6–13.3) months for patients with high PD-L1 expression (Figure S5). Median rwPFS (95% CI) was 4.7 (4.1–5.4) months for patients with non-squamous cell or other histology and 3.9 (3.1–5.0) months for patients with squamous cell histology (Figure S6).

## 4. Discussion

Using data from the IRST in Italy, this real-world study provides insights into patient characteristics, treatment patterns, and survival outcomes for patients initiating a first-line therapy for advanced NSCLC before and after the introduction of newer treatment options (e.g., ICIs and targeted therapies).

Characteristics of the patient population in the current study were comparable to those observed in other European real-world studies performed in the Netherlands, Sweden and the UK [25,26,27]. Data on ECOG PS were available for most of the study population, and the majority of patients with data had an ECOG PS of ≤1. Nevertheless, approximately 18% of the study population had an ECOG PS of ≥2, a characteristic that would typically exclude them from clinical trials. PD-L1 testing was performed for more than half of all patients over the study period, and the proportion of patients with a conclusive PD-L1 test result increased noticeably from 9% in 2016 to 89% in 2018, in alignment with approvals of PD-(L)1 ICIs and associated ESMO guidance on PD-L1 testing [7,28,29,30,31]. In addition, the observed temporal trends of testing for oncogenic driver mutations generally reflected changes to treatment availability (e.g., the European Medicines Agency approvals of crizotinib for *ALK*- and *ROS1*-postive NSCLC in 2015 and 2016, respectively, and alectinib in 2017 and brigatinib in 2018 for *ALK*-positive NSCLC [32,33,34,35]) as well as associated guidance from ESMO on testing for these mutations [7,31].

Chemotherapy was the most frequently recorded first line of therapy for advanced NSCLC across the entire study period; however, temporal variations showed a notable increase in the use of first-line anti-PD-(L)1 ICIs (as monotherapy or combined with chemotherapy) from 2018 onwards, parallel with a decrease in the use of first-line chemotherapy. Moreover, across the study period, anti-PD-(L)1 ICI monotherapy was the most common second-line treatment. Overall, the treatment patterns and sequencing data from this study suggest that anti-PD-(L)1 ICIs are increasingly preferred in real-world practice for the first- and second-line treatment of advanced NSCLC, presumably in patients not harbouring actionable genomic alterations, although second- or later-line use of these agents may decrease with increased first-line use due to guidance on avoiding anti-PD-(L)1 ICI retreatment/rechallenge in certain patient populations [14]. These observations are consistent with results from real-world studies conducted in Canada, Germany, Sweden, the UK and the US over a similar time period, which all showed a rapid and noteworthy uptake of immunotherapy as first-line treatment for patients with advanced NSCLC that generally coincided with approvals/public reimbursement of anti-PD-(L)1 ICIs in the respective countries [26,27,36,37,38]. In addition, the observed rapid uptake of ICIs in our study population builds upon the findings of an earlier analysis conducted at the IRST that looked at treatment patterns and outcomes in patients with advanced NSCLC and no or an unknown oncogene-addicted tumour (*EGFR*-, *ALK*- and *ROS1*-negative or unknown) in pre- and post-immunotherapy time periods (based on immunotherapy availability in the Emilia-Romagna region) [24]. In that earlier analysis by Danesi et al., and as expected, no patients received first-line ICI therapy during the pre-immunotherapy period (January 2014 to June 2017). However, during the post-immunotherapy period (July 2017 to June 2020), 26% of patients received first-line ICIs [24]. The use of targeted therapy also increased during our study, but to a much lesser extent than that seen with immunotherapy; this also mirrors reported treatment patterns in prior real-world studies of patients with advanced NSCLC in Canada and the UK [27,37].

The median OS reported herein for the overall population (8.2 months) was marginally shorter than that reported in prior real-world studies of patients with advanced NSCLC conducted over a similar time period in Canada (10.9 months), Germany (10.9 months), the UK (9.5 months) and the US (10.7 months) [27,38]. However, study- and country-specific variations in survival outcomes are to be expected and can be influenced by a multitude of patient- and healthcare-related factors. Indeed, in a recent multi-country study enrolling patients with advanced NSCLC using standardised inclusion and exclusion criteria across five European countries, median OS fluctuated between a low of 5 months (in France) and a high of 9 months (in Germany) [39]. Over the course of our study, there was a trend towards improved survival outcomes from 2018 onwards, which coincided with a noteworthy increase in the use of anti-PD-(L)1 ICIs in the study population. This observation is consistent with results from the earlier analysis conducted at the IRST showing improvements in both OS and rwPFS in the post-immunotherapy time period versus the pre-immunotherapy time period [24]. Moreover, these findings also align with previous real-world studies showing improved OS in post- versus pre-immunotherapy time periods in advanced NSCLC populations in Canada (12.1 versus 10.2 months), Denmark (11.0 versus 7.8 months) and Germany (14.8 versus 9.4 months) [36,37,40]. While our study was not designed to determine the direct influence of increased first-line use of immunotherapy-based treatments on survival outcomes, it is likely that this treatment evolution contributed to the survival improvements seen over the same timeframe. In the earlier analysis conducted at the IRST, median OS and rwPFS for patients receiving ICIs in the post-immunotherapy period (15.5 and 12.1 months, respectively) was substantially improved over those reported for all patients in the pre-immunotherapy period (6.2 and 3.7 months, respectively) [24]. Likewise, in a previous Danish study, median OS for patients receiving ICIs in the post-immunotherapy period was also substantially improved (19.0 months) over that reported for all patients treated in the pre-immunotherapy period (7.8 months) [40]. It is important to consider that, with widespread PD-L1 expression testing, it has become easier to identify those patients who are more likely to benefit from anti-PD-(L)1 ICI treatment, and, as such, comparisons with pre-immunotherapy “all-comer” populations should be interpreted appropriately. Moreover, it is not possible to discount the contribution of other factors on the observed improved OS outcomes, such as the availability of more effective second- or later-line therapies, general advances in patient management and palliative care, improvements in disease diagnosis, disease stage migrations and/or patient selection bias during this period. For example, OS for patients receiving chemotherapy in the post-immunotherapy period in the Danish study was also improved over that reported for all patients treated in the pre-immunotherapy period, albeit to a lesser extent than seen with ICIs [40]. The authors speculated that this was a result of several factors, including subsequent ICI treatment, stage migration owing to improved staging diagnostics, improved palliative care, changes in histopathological subtypes and advances in molecular testing [40].

Survival outcomes were improved among patients with recurrent disease versus those with a de novo diagnosis, a pattern that was also observed in prior studies of advanced NSCLC populations in Canada and South Korea [41,42,43]. Although the reason for this difference has not been defined, a suggested contributing factor is a greater tumour burden among patients with de novo disease, potentially caused by less routine monitoring of this population and a resultant higher frequency of extrapulmonary metastatic sites [42,43]. To support this hypothesis, in the current study, the proportion of patients with metastases in pulmonary sites (i.e., contralateral lung and pleura) was relatively similar among those with a de novo diagnosis or recurrent disease; however, the proportion of patients extrapulmonary metastases (i.e., in bone, lymph nodes and liver) was noticeably higher among those with a de novo diagnosis versus those with recurrent disease. Treatment received after recurrent or de novo disease diagnosis may have also been a contributor to the differing survival outcomes. A higher proportion of patients with recurrent disease received ICIs and targeted therapies versus chemotherapy at both the first line and second line. We also cannot exclude other baseline characteristics or lead-time bias as potential contributors to the differing survival outcomes.

Median OS and rwPFS were longer for patients receiving first-line targeted therapy than those receiving anti-PD-(L)1 ICIs (either as monotherapy or with chemotherapy) or chemotherapy alone. While we were unable to confirm the exact proportion due to data masking, the available data indicated that between 89% and 91% of the patients receiving first-line targeted therapy in this study tested positive for *EGFR*, *ALK* or *ROS1* mutations, based on the premise that dual positive mutations are extremely rare in patients with NSCLC [44,45,46]. Moreover, as some of the patients received TKIs targeting other mutations for which data on testing patterns were not collected (e.g., dabrafenib plus trametinib, which targets tumours with a *BRAF* V600E mutation), it is assumed that all patients receiving targeted therapy did so due to a positive mutational test result. On this basis, the observed longer OS and rwPFS among those receiving targeted therapy were almost certainly due to the use of precision medications that are specifically designed for each actionable genomic alteration [47]. When stratified by tumour PD-L1 expression, both median OS and median rwPFS were longest in patients with high expression (≥50%), of which around three-quarters received first-line anti-PD-(L)1 ICI monotherapy. This observation aligns with a previous meta-analysis showing that patients with NSCLC and accompanying high PD-L1 expression tended to achieve increased benefit from treatment with PD-(L)1 inhibitors [48]. Interestingly, in a recent real-world analysis of patients with advanced NSCLC receiving induction therapy with pembrolizumab plus chemotherapy across five European countries, tumour PD-L1 expression had no clear impact on OS or PFS outcomes [39]. However, other studies have shown improved survival with pembrolizumab plus pemetrexed versus pembrolizumab monotherapy in patients with low-to-moderate PD-L1 expression [49], which may explain why PD-L1 expression has limited impact on outcomes with combinations of pembrolizumab and chemotherapy.

The difference between median OS and rwPFS was less clear between anti-PD-(L)1 ICIs as monotherapy and in combination with chemotherapy. The median time was numerically longer for patients receiving anti-PD-(L)1 ICI monotherapy than those receiving anti-PD-(L)1 ICI with chemotherapy; however, the difference was slight, with confidence intervals of the medians overlapping and a marginal separation in the survival curves apparent only after 6–12 months. Thus, no firm conclusions can be drawn between treatment with anti-PD-(L)1 ICI monotherapy and anti-PD-(L)1 ICI with chemotherapy.

Median OS and rwPFS were longer in patients with non-squamous cell or other histology versus those with squamous cell histology. This was perhaps expected since longer OS has frequently been reported among treated patients with non-squamous versus squamous NSCLC in clinical trials and real-world studies [50,51,52,53,54,55]. Moreover, because most of the actionable genomic alterations occur in non-squamous cell carcinomas (predominantly adenocarcinomas) and, consequently, ESMO guidelines do not recommend general molecular testing in patients with squamous cell carcinomas [7,15,31,56], survival outcomes among patients with non-squamous cell or other histology in the current study were likely further enhanced by the use of targeted therapy in these patients.

The strengths of the current study included the large population of patients initiating a first-line therapy for advanced NSCLC; the capture of treatment and survival data across almost a decade (2014 to 2021); the inclusion of patients who might otherwise not be eligible for clinical trials due to factors such as advanced age and poor PS; and the use of hospital EHRs that allowed for the collection of comprehensive patient data (including those for disease staging, PS and biomarker testing) and real-world survival data (including rwPFS). Limitations of the study were as follows: (i) the analyses were restricted to patients at the IRST, which, being a single-centre hospital-based population, may not be fully representative of patient populations at other cancer centres across the Emilia-Romagna region or Italy as a whole; (ii) the analyses excluded patients enrolled in clinical trials, which may have introduced a selection bias as these patients may have had different characteristics than those not enrolled in clinical trials; (iii) the analyses were limited only to treated patients; (iv) the accessed EHRs may not have fully captured all data of interest; (v) the treatment categories and treatment sequences were determined algorithmically and, as such, there is an inherent potential for misclassification bias; (vi) some of the subgroup analyses were restricted by sample size, potentially limiting data interpretation; (vii) rwPFS relied partially on physician-recorded progression, which is subject to variability; (viii) survival outcomes were not statistically tested or adjusted for confounding variables or conducted using multivariable methods limiting causal interpretation; (ix) dedicated survival analyses for patients carrying *EGFR*, *ALK* or *ROS1* mutations/alterations were not conducted and thus the benefit of targeted therapies on the individual mutations cannot be determined; (x) testing of PD-L1 expression was limited, predominately in the initial years of the study, potentially biassing the observed survival outcomes and (xi) survival outcomes may have been impacted by the evolving standards of care and the COVID-19 pandemic. Evolving standards of care include improvements in disease diagnosis, availability of more effective therapies, and general advances in patient management and palliative care. The COVID-19 pandemic in 2020 and 2021 may have impacted the number of patients receiving treatment, the types of treatment administered, and hence, the observed survival outcomes. During this time, the IRST implemented a COVID-free policy, allowing patients with cancer to continue their treatments in a controlled, safe environment free from risks associated with the pandemic. As a result, the cohort may have included healthier patients with fewer comorbidities choosing to access care at the IRST, which may have contributed to the higher median overall survival observed in 2020 compared with other study years.

## 5. Conclusions

This study provides real-world insights into the changing treatment landscape for patients with advanced NSCLC, with a notable pattern of increased use of first-line anti-PD-(L)1 ICIs from 2018 onwards, which aligns with their approval in the first-line setting. Overall, while prognosis for patients with advanced NSCLC is typically poor, results from this study show that patients with actionable genomic alterations continue to benefit from treatment with precision targeted therapies and that the approval of anti-PD-(L)1 ICIs has further evolved the treatment paradigm for patients with advanced NSCLC.

## Figures and Tables

**Table 1:** Patient characteristics for the overall population and by diagnosis type.

	Overall Population (N = 910)	De Novo Diagnosis (n = 758)	Recurrent Disease (n = 152)
**Age, years ^a^**			
Median (IQR)	69.9 (63.3–76.0)	69.7 (62.8–75.7)	71.3 (66.2–77.3)
<75	652 (72%)	547 (72%)	105 (69%)
**Sex, male ^b^**	556 (61%)	459 (61%)	97 (64%)
**Tumour stage** **at initial NSCLC diagnosis**			
I	44 (5%)	0	44 (29%)
II/III	148 (16%)	40 (5%)	108 (71%)
IV	718 (79%)	718 (95%)	0
**Time from initial NSCLC diagnosis to start of first line of therapy for advanced NSCLC**			
Median (IQR), months	1.4 (0.9–2.7)	1.2 (0.8–1.7)	19.8 (9.4–34.9)
**Time from advanced NSCLC diagnosis to start of first line of therapy for advanced NSCLC**			
Median (IQR), months	1.2 (0.8–2.1)	1.2 (0.8–1.7)	2.8 (0.0–10.0)
**Tumour stage** **at study inclusion date**			
IIIA	*	*	*
IIIB	17 (2%)	**	**
IIIC	*	0	*
IV	890 (98%)	750 (99%)	140 (92%)
**Year of first line of therapy initiation for advanced NSCLC**			
2014	106 (12%)	92 (12%)	14 (9%)
2015	101 (11%)	89 (12%)	12 (8%)
2016	116 (13%)	95 (13%)	21 (14%)
2017	112 (12%)	95 (13%)	17 (11%)
2018	126 (14%)	96 (13%)	30 (20%)
2019	125 (14%)	109 (14%)	16 (11%)
2020	115 (13%)	92 (12%)	23 (15%)
2021	109 (12%)	90 (12%)	19 (13%)
**ECOG PS ^a^**			
0	148 (16%)	100 (13%)	48 (32%)
1	570 (63%)	490 (65%)	80 (53%)
2	148 (16%)	139 (18%)	9 (6%)
3	19 (2%)	19 (3%)	0 (0%)
4	0 (0%)	0 (0%)	0 (0%)
Missing	25 (3%)	10 (1%)	15 (10%)
**Histology ^b^**			
Non-squamous cell NSCLC Squamous cell NSCLC Others	736 (81%) 133 (15%) 41 (5%)	619 (82%) ** **	117 (77%) ** *
**Recorded metastases ^b,c^** Bone Contralateral lung Lymph nodes Pleura Liver Other	239 (26%) 238 (26%) 150 (16%) 106 (12%) 75 (8%) 146 (16%)	215 (28%) 198 (26%) 134 (18%) 92 (12%) ** 135 (18%)	24 (16%) 40 (26%) 16 (11%) 14 (9%) * 11 (7%)
**Tumour PD-L1 expression**			
Conclusive test result Low expression (<1%) ^d^	510 (56%) 203 (40%)	421 (56%) 167 (40%)	89 (59%) 36 (40%)
Medium expression (≥1–49%) ^d^	181 (35%)	151 (36%)	30 (34%)
High expression (≥50%) ^d^	126 (25%)	103 (24%)	23 (26%)
Inconclusive test result/not tested	400 (44%)	337 (44%)	63 (41%)
***EGFR*** **mutation status**			
Conclusive test result	705 (77%)	593 (78%)	112 (74%)
Positive ^d^	119 (17%)	101 (17%)	18 (16%)
Negative ^d^	586 (83%)	492 (83%)	94 (84%)
Inconclusive test result/not tested	205 (23%)	165 (22%)	40 (26%)
***ALK*** **mutation status**			
Conclusive test result	644 (71%)	541 (71%)	103 (68%)
Positive ^d^	41 (6%)	34 (6%)	7 (7%)
Negative ^d^	603 (94%)	507 (94%)	96 (93%)
Inconclusive test result/not tested	266 (29%)	217 (29%)	49 (32%)
***ROS1*** **mutation status**			
Conclusive test result	476 (52%)	400 (53%)	76 (50%)
Positive ^d^	7 (1%)	**	*
Negative ^d^	469 (99%)	**	**
Inconclusive test result/not tested	434 (48%)	358 (47%)	76 (50%)

Categorical data are shown as amount (percentage). Total percentages may not equal 100% due to rounding. * Indicates primary data masking of patient counts between 1 and 4; ** indicates secondary data masking to prevent calculation of primary masked data. ^a^ Recorded at inclusion date (i.e., start of a non-curative first line of therapy at the IRST). ^b^ Recorded at diagnosis of advanced NSCLC. ^c^ Patients could have more than one metastatic site. ^d^ Expressed as a percentage of patients with a conclusive test result. *ALK*: anaplastic lymphoma kinase; ECOG PS: Eastern Cooperative Oncology Group performance status; *EGFR*: epidermal growth factor receptor; IQR: interquartile range; IRST: IRCCS Istituto Romagnolo per lo Studio dei Tumori; LoT: line of therapy; NSCLC: non-small-cell lung cancer; PD-L1: programmed death ligand 1; *ROS1*: c-ros oncogene 1 receptor tyrosine kinase.

**Table 2:** Treatment patterns for the overall population and by diagnosis type.

	Overall Population (N = 910)	De Novo Diagnosis (n = 758)	Recurrent Disease (n = 152)
**First-line treatment for advanced NSCLC**			
Chemotherapy ^a^ Platinum-based chemotherapy	566 (62%) 370 (41%)	494 (65%) 337 (44%)	72 (47%) 33 (22%)
Non-platinum-based chemotherapy	196 (22%)	157 (21%)	39 (26%)
Anti-PD-(L)1 ICI Anti-PD-(L)1 ICI monotherapy ^b^ Anti-PD-(L)1 ICI + chemotherapy ^c^	** 122 (13%) **	** 81 (11%) **	** 41 (27%) **
Targeted therapy ^d^ Other treatments ^e^	149 (16%) *	118 (16%) *	31 (20%) *
**Median duration of first-line treatment, weeks (IQR)**	13.4 (7.2–30.4)	13.2 (6.7–30.1)	15.0 (8.4–38.2)
**No. of lines of therapy**			
1	584 (64%)	491 (65%)	93 (61%)
≥2	326 (36%)	267 (35%)	59 (39%)
≥3	123 (14%)	101 (13%)	22 (14%)
≥4	46 (5%)	38 (5%)	8 (5%)
**Immunotherapy during study ^f^**	342 (38%)	263 (35%)	79 (52%)

Categorical data are shown as amount (percentage). Total percentages may not equal 100% due to rounding. * Indicates primary data masking of patient counts between 1 and 4; ** indicates secondary data masking to prevent calculation of primary masked data. ^a^ Includes singlet, doublet, etc., chemotherapy regimens; does not include combinations of chemotherapy with other classes of treatment. ^b^ Includes single-agent ICIs. ^c^ Includes combinations of ICIs with chemotherapy; does not include combinations of ICIs. ^d^ Includes single-agent TKIs. ^e^ Includes combinations of TKIs or other monoclonal antibodies with other classes of treatment (e.g., chemotherapy). ^f^ Represents treatment with an anti-PD-(L)1 ICI (as monotherapy or in combination with chemotherapy) at any line of therapy. ICI: immune checkpoint inhibitor; IQR: interquartile range; PD-(L)1: programmed death (ligand) 1; NSCLC: non-small-cell lung cancer; TKI: tyrosine kinase inhibitor.

## Data Availability

Data presented in this article are not publicly available, and no data sharing is planned. Patient-level data cannot to be shared due to relevant regulatory and confidentiality reasons. All data are presented in aggregate form only.

## Supplementary Materials

The following supporting information can be downloaded: https://www.mdpi.com/article/10.3390/cancers18030538/s1. Table S1: Algorithm to determine treatment intent of first treatment recorded for stage IIIA/IIIB patients; Table S2: Temporal testing patterns for PD-L1 expression and key actionable genomic alterations in the overall population; Figure S1: Treatment sequencing (first to fourth line) for patients with a de novo diagnosis; Figure S2: Treatment sequencing (first to fourth line) for patients with recurrent disease; Figure S3: Overall survival for the overall population stratified by tumour PD-L1 status; Figure S4: Overall survival for the overall population stratified by histology; Figure S5: Real-world progression-free survival for the overall population stratified by tumour PD-L1 status; Figure S6: Real-world progression-free survival for the overall population stratified by histology.
