# Supplementary material for: Real-World Treatment Patterns and Survival in Patients with Advanced Non-Small Cell Lung Cancer: An Italian Retrospective Cohort Study
Source: Cancers (Basel). 2026 Feb 6;18(3):538. doi: 10.3390/cancers18030538 (PMC12897191; doi:10.3390/cancers18030538)
Supplement: Supplementary file 1 [file cancers-18-00538-s001.zip › cancers-4074043-supplementary.pdf]

cancers

MDPI

Supplementary Materials: Real-World Treatment Patterns and Survival in Patients with Advanced Non-Small Cell Lung Cancer: An Italian Retrospective Cohort Study

Angelo Delmonte, Nicola Gentili, Andrea Roncadori, Roberta Maltoni, Valentina Danesi, Pooja Hindocha, Cátia Leal, Stavros Oikonomou, Marta Mella, Sarah Lay-Flurrie, Gabrielle Emanuel, Caroline Rault, Mrudula B. Glassberg, Adam Lee, Yong Yuan and Ilaria Massa

Table S1. Algorithm to determine treatment intent of first treatment recorded for stage IIIA/IIIB patients.

**Algorithm step**		**Intent of SACT**
**Consider first SACT regimen at of time entry in IRST**		
1. Did the SACT regimen include immunotherapy?	Yes—*go to next step*	
	No—*go to step 3*	
2. Did the SACT regimen include durvalumab?	Yes	Curative
	No	Advanced
3. Did the SACT regimen include a TKI?	Yes	Advanced
	No—*go to next step*	
**The SACT regimen does not include any of the above**		
4. Was the SACT regimen part of a chemoradiation schedule (radiotherapy + SACT, concurrent or sequential, with or without durvalumab)?	Yes	Curative
	No—*go to next step*	
5. Was the SACT regimen adjuvant to surgery?	Yes	Curative
	No—*go to next step*	
6. Was the SACT regimen intended to be neoadjuvant to surgery?	Yes—information on intent retrieved from notes	Curative
	Yes—there is a record of surgery after SACT	Curative
	No—*go to next step*	
7. Do treatment notes state SACT regimen was administered with advanced intent?	Yes	Advanced
	No—*go to next step*	
8. Do treatment notes state SACT regimen was administered with curative intent?	Yes	Curative
	No—*go to next step*	
9. Do treatment notes NOT state SACT regimen intent?	Yes—*go to next step*	
10. Was the SACT regimen part of a schedule including radiotherapy, but not part of a chemoradiation schedule?	Yes for radiotherapy – No information on intent	Assume:<br>Stage IIIA: curative<br>Stage IIIB: advanced
	No for radiotherapy—*go to step 11*	
11. Was the SACT regimen administered as a schedule on its own (i.e., no radiotherapy or surgery)?	Yes (SACT alone)	Advanced

SACT includes chemotherapy, targeted treatments, immunotherapy.

IRST: IRCCS Istituto Romagnolo per lo Studio dei Tumori; SACT: systemic anti-cancer therapy; TKI: tyrosine kinase inhibitor.

https://doi.org/10.3390/cancers18030538

Table S2. Temporal testing patterns for PD-L1 expression and key actionable genomic alterations in the overall population.

	**Year of first line of therapy initiation**							
	**2014 (n = 106)**	**2015 (n = 101)**	**2016 (n = 116)**	**2017 (n = 112)**	**2018 (n = 126)**	**2019 (n = 125)**	**2020 (n = 115)**	**2021 (n = 109)**
**Tumour PD-L1 expression**								
Conclusive test result	*	*	11 (9%)	50 (45%)	112 (89%)	118 (94%)	**	101 (93%)
Inconclusive test result/ not tested	**	**	105 (91%)	62 (55%)	14 (11%)	7 (6%)	*	8 (7%)
***EGFR* mutations**								
Conclusive test result	81 (76%)	79 (78%)	93 (80%)	81 (72%)	103 (82%)	94 (75%)	92 (80%)	82 (75%)
Inconclusive test result/ not tested	25 (24%)	22 (22%)	23 (20%)	31 (28%)	23 (18%)	31 (25%)	23 (20%)	27 (25%)
***ALK* mutations**								
Conclusive test result	46 (43%)	62 (61%)	85 (73%)	***	98 (78%)	***	***	88 (81%)
Inconclusive test result/ not tested	60 (57%)	39 (39%)	31 (27%)	**	28 (22%)	**	**	21 (19%)
***ROS1* mutations**								
Conclusive test result	11 (10%)	16 (16%)	27 (23%)	47 (42%)	***	***	***	87 (80%)
Inconclusive test result/ not tested	95 (90%)	85 (84%)	89 (77%)	65 (58%)	**	**	**	22 (20%)

Categorical data are shown as amount (percentage). *Indicates primary data masking of patient counts between 1 and 4; **indicates secondary data masking to prevent calculation of primary masked data; ***indicates undeterminable patient counts due to masking of a component of the total number of patients (i.e., due to primary data masking of the number of patients with a positive test result, it was not possible to determine the total number of patients with quantifiable data). *ALK*, anaplastic lymphoma kinase; *EGFR*, epidermal growth factor receptor; PD-L1, programmed cell ligand 1; *ROS1*: c-ros oncogene 1 receptor tyrosine kinase.

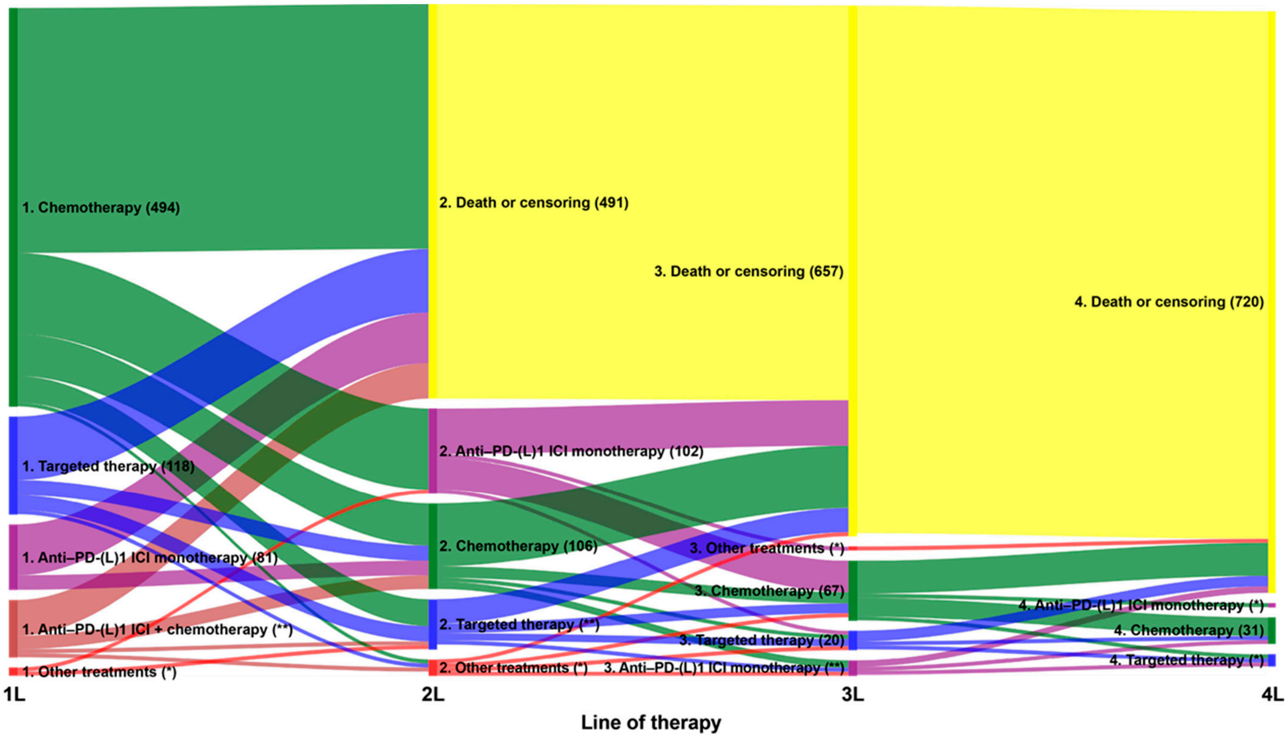

Figure S1. Treatment sequencing (first to fourth line) for patients with a de novo diagnosis. * Indicates primary data masking of patient counts between 1 and 4; ** indicates secondary data masking to prevent calculation of primary masked data. ICI: immune checkpoint inhibitor; PD-(L)1: programmed death (ligand) 1.

https://doi.org/10.3390/cancers18030538

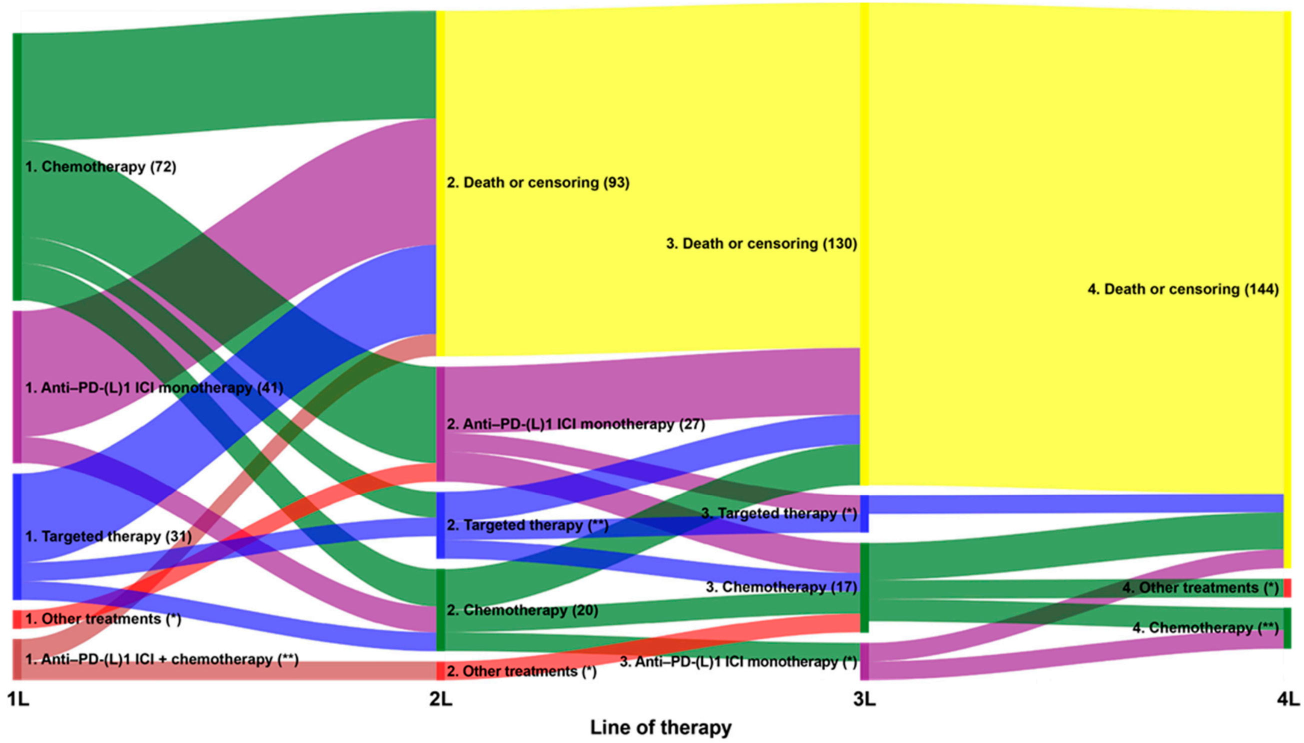

Figure S2. Treatment sequencing (first to fourth line) for patients with recurrent disease. * Indicates primary data masking of patient counts between 1 and 4; ** indicates secondary data masking to prevent calculation of primary masked data. ICI: immune checkpoint inhibitor; PD-(L)1: programmed death (ligand) 1;

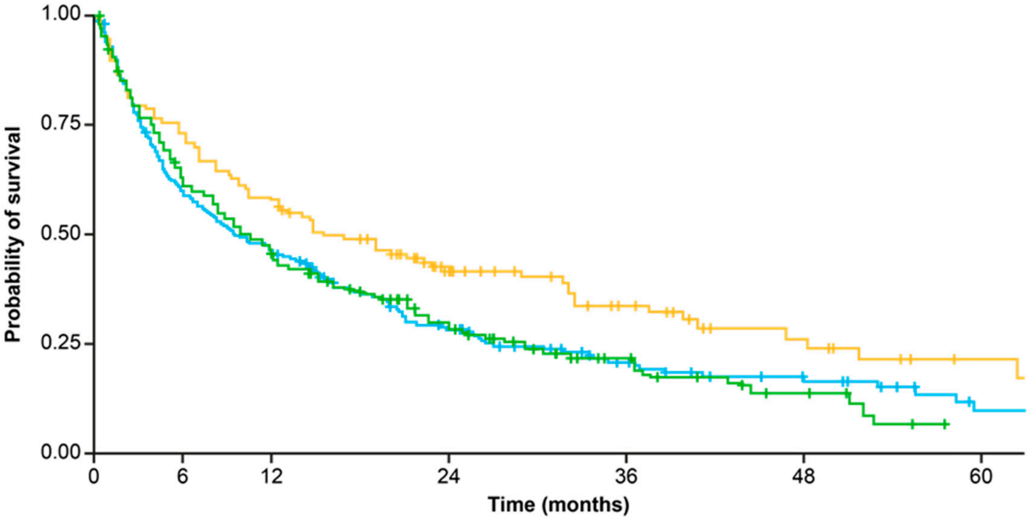

No. at risk	0	6	12	24	36	48	60
Low (<1% or negative)	203	119	93	48	27	15	*
Medium (1–49%)	181	111	80	39	16	6	0
High (≥50%)	126	92	73	39	22	12	*

PD-L1 status	Number of patients	Median OS, months (95% CI)	Survival probability (95% CI)[a]	
			1 year	3 years
Low (<1% or negative)	203	9.4 (7.0–14.7)	0.46 (0.40–0.54)	0.21 (0.16–0.28)
Medium (1–49%)	181	10.6 (8.2–14.5)	0.46 (0.39–0.54)	0.22 (0.16–0.30)
High (≥50%)	126	15.5 (11.9–31.7)	0.58 (0.50–0.67)	0.34 (0.26–0.44)

Figure S3. Overall survival for the overall population stratified by tumour PD-L1 status. [a] Survival probabilities are suppressed when the number of patients at risk is <10. * Indicates primary data masking of patient counts between 1 and 4. CI: confidence interval; PD-L1: programmed death ligand 1; OS: overall survival.

https://doi.org/10.3390/cancers18030538

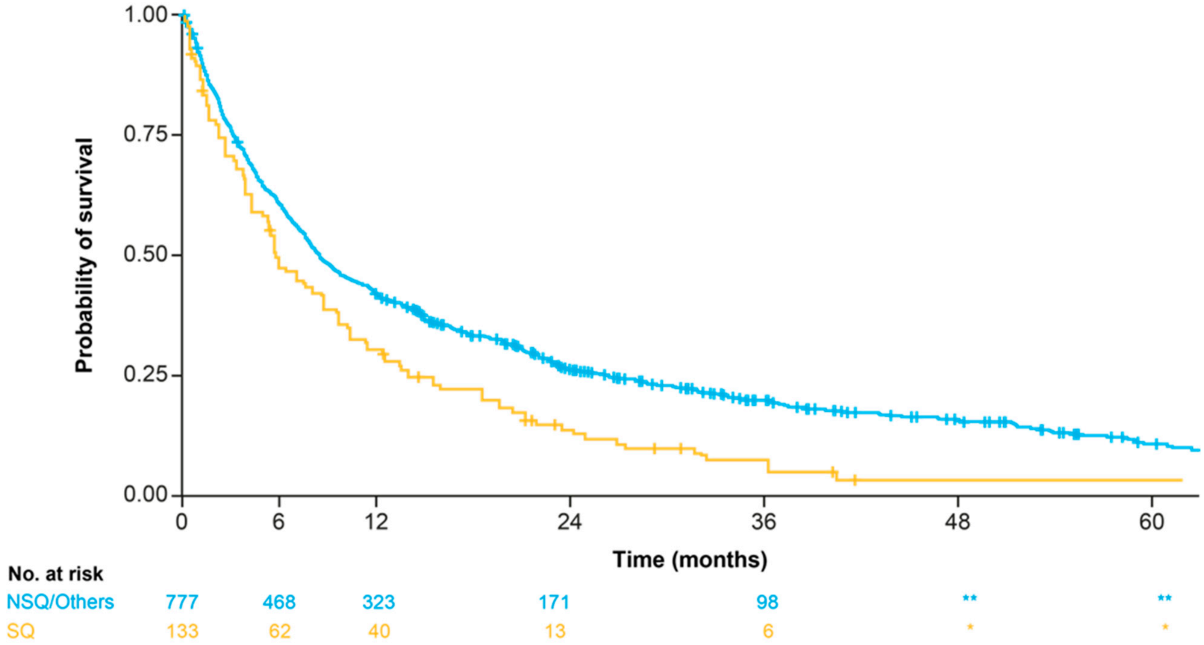

Histology	Number of patients	Median OS, months (95% CI)	Survival probability (95% CI)[a]	
			1 year	3 years
NSQ/Others	777	8.5 (7.7–9.7)	0.42 (0.39–0.46)	0.20 (0.17–0.23)
SQ	133	5.8 (5.2–8.8)	0.31 (0.24–0.40)	—

Figure S4. Overall survival for the overall population stratified by histology. * Indicates primary data masking of patient counts between 1 and 4; ** indicates secondary data masking to prevent calculation of primary masked data. [a] Survival probabilities are suppressed when the number of patients at risk is <10. CI: confidence interval; NSQ: non-squamous; OS: overall survival; SQ: squamous.

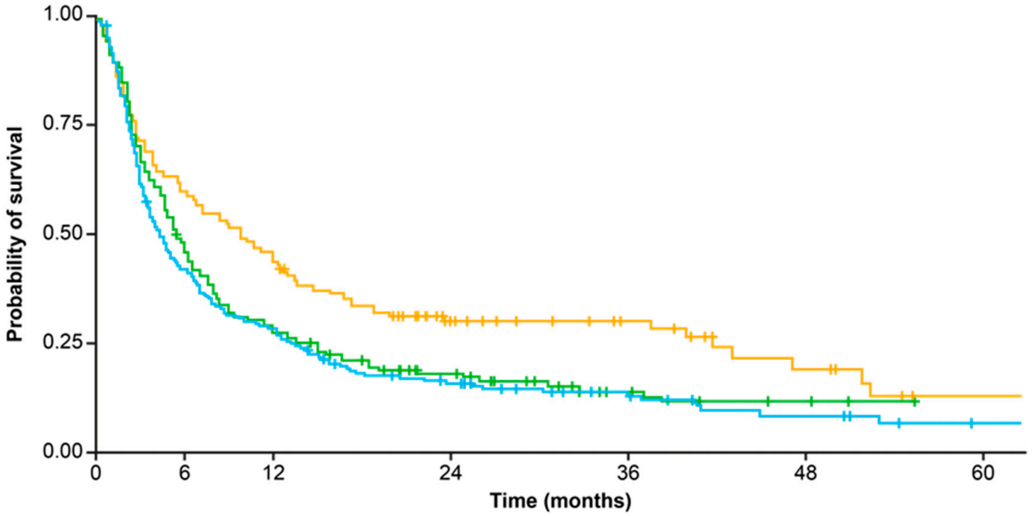

No. at risk	0	6	12	24	36	48	60
Low (<1% or negative)	203	84	57	27	15	**	*
Medium (1–49%)	181	81	49	23	7	*	0
High (≥50%)	126	76	58	27	17	8	*

PD-L1 status	Number of patients	Median rwPFS, months (95% CI)	Survival probability (95% CI)[a]	
			1 year	3 years
Low (<1% or negative)	203	4.3 (3.5–5.6)	0.28 (0.23–0.35)	0.13 (0.09–0.19)
Medium (1–49%)	181	5.5 (4.7–6.6)	0.28 (0.22–0.35)	—
High (≥50%)	126	9.8 (6.6–13.3)	0.46 (0.38–0.56)	0.30 (0.23–0.40)

Figure S5. Real-world progression-free survival for the overall population stratified by tumour PD-L1 status. * Indicates primary data masking of patient counts between 1 and 4; ** indicates secondary data masking to prevent calculation of primary masked data. [a] Survival probabilities are suppressed when the number of patients at risk is <10. CI: confidence interval; PD-L1: programmed death ligand 1; rwPFS: real-world progression-free survival.

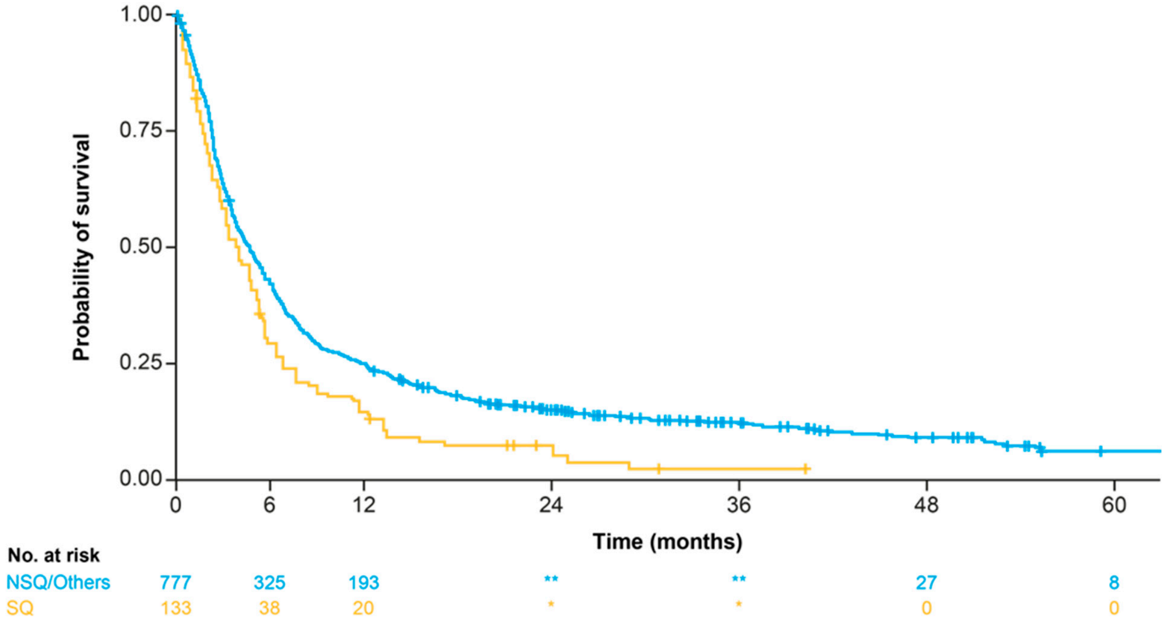

Histology	Number of patients	Median rwPFS, months (95% CI)	Survival probability (95% CI)[a]
			1 year
NSQ/Others	777	4.7 (4.1–5.4)	0.25 (0.22–0.28)
SQ	133	3.9 (3.1–5.0)	0.16 (0.10–0.23)

Figure S6. Real-world progression-free survival for the overall population stratified by histology. * Indicates primary data masking of patient counts between 1 and 4; ** indicates secondary data masking to prevent calculation of primary masked data. [a] Survival probabilities are suppressed when the number of patients at risk is <10. CI: confidence interval; NSQ: non-squamous; rwPFS: real-world progression-free survival; SQ: squamous.
